# An NIH investment in health equity - the economic impact of the Flint Center for Health Equity Solutions

**DOI:** 10.1186/s12889-021-11795-5

**Published:** 2021-09-29

**Authors:** Cristian I. Meghea, Barrett Wallace Montgomery, Roni Ellington, Ling Wang, Clara Barajas, E. Yvonne Lewis, Sheridan T. Yeary, Laurie A. Van Egeren, Debra Furr-Holden

**Affiliations:** 1 Department of Obstetrics, Gynecology and Reproductive Biology, Michigan State University, East Lansing, MI 48824-1316 USA; 2 Department of Epidemiology and Biostatistics, Michigan State University, East Lansing, MI 48824-1316 USA; 3 Department of Advanced Studies, Leadership, and Policy, Morgan State University, Baltimore, MD 21214 USA; 4 Department of Medicine, Michigan State University, East Lansing, MI 48824-1316 USA; 5 Division of Public Health, Michigan State University, Flint, MI 48502 USA; 6 National Center for African American Health Consciousness, Flint, MI 48501 USA; 7 College of Public Affairs, University of Baltimore, Baltimore, MD 21201 USA; 8 University Outreach and Engagement, Michigan State University, East Lansing, MI 48824-1316 USA

**Keywords:** Health disparities, Economic impact, Return on investment

## Abstract

**Background:**

Health disparities are pervasive and are linked to economic losses in the United States of up to $135 billion per year. The Flint Center for Health Equity Solutions (FCHES) is a Transdisciplinary Collaborative Center for health disparities research funded by the National Institute of Minority Health and Health Disparities (NIMHD). The purpose of this study was to estimate the economic impact of the 5-year investment in FCHES in Genesee County, Michigan.

**Methods:**

The estimated impacts of FCHES were calculated using a U.S.-specific input/output (I/O) model, IMPLAN, from IMPLAN Group, LLC., which provides a software system to access geographic specific data regarding economic sector interactions from a variety of sources. This allowed us to model the cross-sector economic activity that occurred throughout Genesee County, Michigan, as a result of the FCHES investment. The overall economic impacts were estimated as the sum of three impact types: 1. Direct (the specific expenditures impact of FCHES and the Scientific Research and Development Services sector); 2. Indirect (the impact on suppliers to FCHES and the Scientific Research and Development Services sector); and 3. Induced (the additional economic impact of the spending of these suppliers and employees in the county economy).

**Results:**

The total FCHES investment amounted to approximately $11 million between 2016 and 2020. Overall, combined direct, indirect, and induced impacts of the total FCHES federal investment in Genesee County included over 161 job-years, over $7.6 million in personal income, and more than $19.2 million in economic output. In addition, this combined economic activity generated close to $2.3 million in state/local and federal tax revenue. The impact multipliers show the ripple effect of the FCHES investment. For example, the overall output of over $19.2 million led to an impact multiplier of 1.75 – every $1 of federal FCHES investment led to an additional $.75 of economic output in Genesee County.

**Conclusions:**

The FCHES research funding yields significant direct economic impacts above and beyond the direct NIH investment of $11 million. The economic impact estimation method may be relevant and generalizable to other large research centers such as FCHES.

## Background

Health disparities are pervasive and are linked to economic losses of up to $135 billion per year, including $93 billion in excess medical care costs and $42 billion in missed productivity [1]. A Health Inequities Think Tank convened by the National Institutes of Health (NIH) agreed that “confronting health inequities will require engaging multiple disciplines and sectors (including communities), using systems science, and intervening through combinations of individual, family, provider, health system, and community-targeted approaches” [2]. The Think Tank issued a set of recommendations for reducing health inequities in the United States, which included embracing broad and inclusive research themes that incorporate multilevel factors; developing research platforms for innovative transdisciplinary research that promote systems science approaches; developing networks of collaborators and stakeholders, and launching transformative studies that can serve as benchmarks; optimizing the use of new data sources, platforms, and natural experiments; and developing unique transdisciplinary training programs to build research capacity.

NIH, through the National Institute on Minority Health and Health Disparities, established the Transdisciplinary Collaborative Centers for Health Disparities Research Program (TCC). TCC supports regional coalitions of academic institutions, community organizations, health providers and systems, and other stakeholders. Current TCC research program grants focus on multilevel chronic disease prevention research, health policy research, social determinants of health, men’s health research, and precision medicine research. As health and disparities are affected by diverse public and private stakeholders, addressing health disparities requires a transdisciplinary approach and strong collaborations between researchers and community organizations, health providers and systems, government agencies, and other stakeholders. Such an approach ensures that findings translate into sustainable changes at multiple levels to reduce inequities in health and improve population health.

The Flint Center for Health Equity Solutions (FCHES) is a Transdisciplinary Collaborative Center (TCC) for health disparities research funded by NIMHD. FCHES focuses its research efforts on health disparities and chronic disease that cross boundaries and directly affect the Flint and Genesee County Community. FCHES is an assembly of stakeholders, including public health researchers, policymakers, health officials, community organizations, and faith-based partners across a range of specialties to mount evidenced-based and promising approaches to prevent chronic disease and reduce health inequities.

**Fig. 1:**
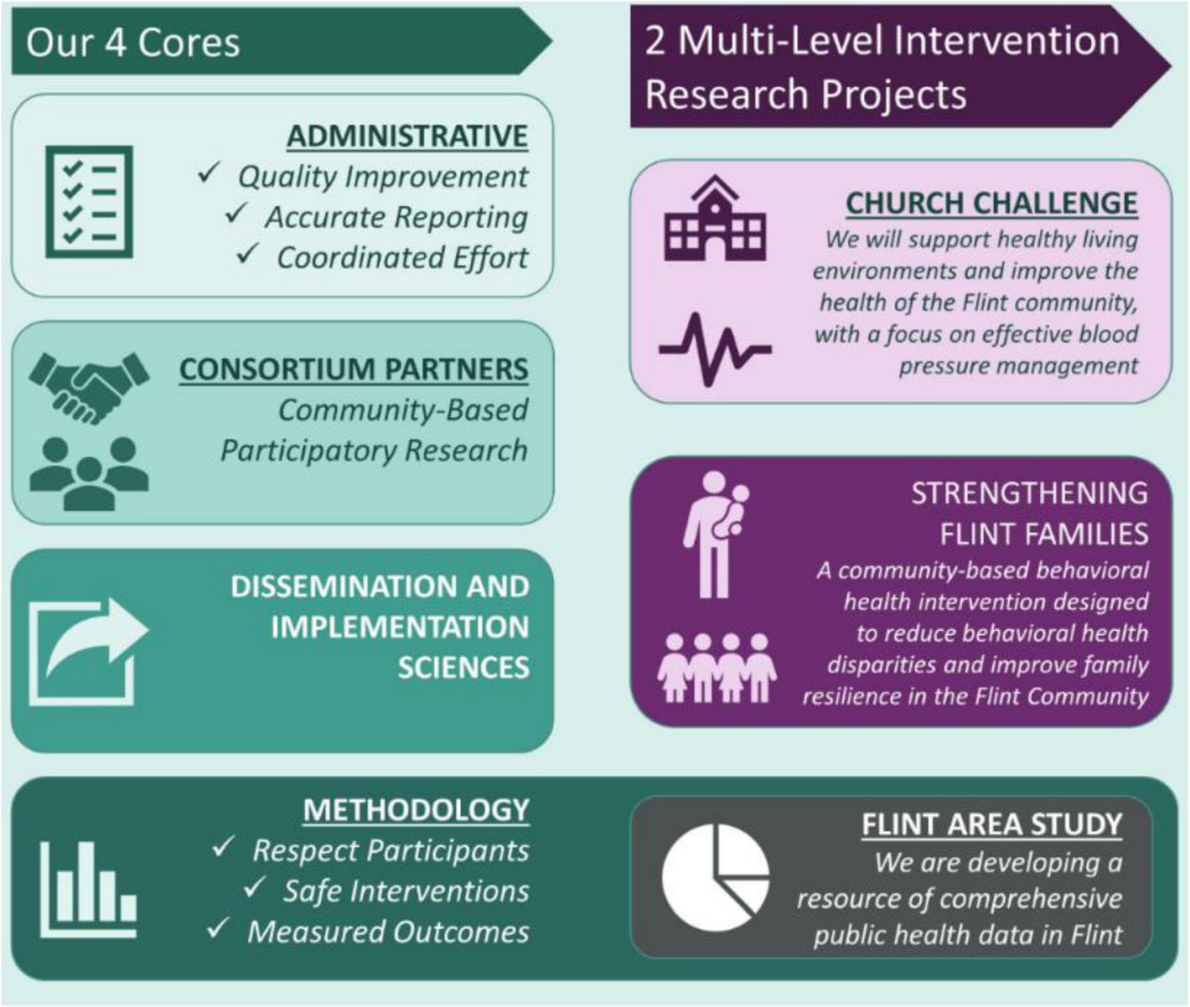
The structure of FCHES – Flint Center for Health Equity Solutions

Figure 1 presents the structure of FCHES, which includes four cores, two multilevel intervention research projects, and the Flint Area Study, a comprehensive resource of public health data in Flint developed by FCHES. This study will provide analyses and report on the short-term direct and indirect economic impacts in Genesee County, MI resulting from the expenditures in FCHES; examples and case studies of functional impacts of FCHES; and a brief discussion of the potential medium- and long-term economic impacts of FCHES in other MI counties and statewide. The economic impact is not the only measure of success for FCHES. Another study [3] is presenting the framework for the overall FCHES evaluation.

The economic impact analysis reported in this study uses an input/output (I/O) model to represent the interrelationships among economic sectors [4, 5]. I/O models are quantitative economic models representing the interdependencies between different sectors of a national or regional economy (in this case Genesee County, MI). Historically, various approaches were used to estimate the economic impact of research, education, businesses, projects, policy, or economic events. Economic impact methods include subjective estimates relying on expert opinion, secondary data, or primary data and formal models involving surveys and regional economic models [6]. The first two approaches are hampered by the somewhat subjective nature of expert opinions and the general limitations of secondary data, including the fact that the data were collected for other initial purposes and may not fit the particular needs of the analysis. Most major economic impact models in the recent years, including studies estimating impacts of research funding [7, 8] similar to this one, use I/O estimation methods. The main advantages of using I/O models include the fact that they account for the interdependent nature of the sectors in the economy and can estimate impacts on various sectors caused by changes in one sector [9], and the fact that they rely on current, complete, data sources such as the Bureau of Labor Statistics, the Bureau of Economic Analysis, and the Census Bureau. I/O models, such as IMPLAN (details below) are widely used to evaluate economic impacts. The theoretical I/O framework used by IMPLAN was developed by Wassily Leontief, for which he received the Nobel Prize in 1973.

The purpose of this current study was to estimate the economic impact of the 5-year NIH research investment in FCHES in Genesee County, Michigan and to provide an economic evaluation model potentially replicable by other TCCs and other large research centers. Relevant for the perspective of the results and the discussion in this study, the mission of FCHES and other similar large research centers is to conduct public health research.

## Methods

**Fig. 2:**
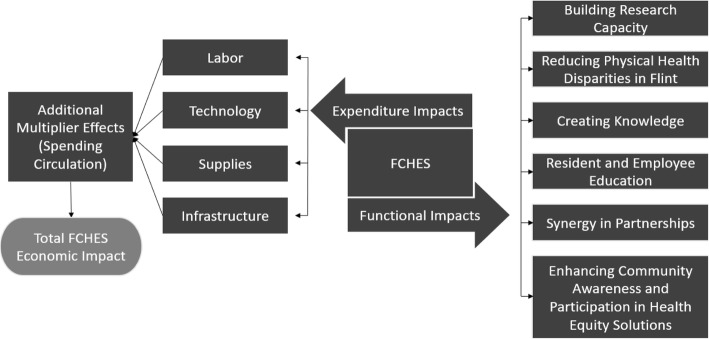
The structure of the impacts associated with FCHES

Figure 2 illustrates the connections between the direct investments in the FCHES and the impacts associated with these investments.

The estimated impacts of FCHES were calculated using a U.S.-specific I/O model, IMPLAN, from IMPLAN Group, LLC., which provides a software system to access geographic specific data regarding economic sector interactions from a variety of sources. Data from U.S. government sources (e.g. Bureau of Economic Analysis, Department of Agriculture, Bureau of Labor Statistics, Census, and others) are collected and merged on an annual basis to produce a complete set of Social Accounting Matrices (SAMs) for each sector and region of interest. If the data source is not updated annually, the IMPLAN data team estimates the missing pieces and includes this estimate in the annual updates. The SAMs provide a complete picture of an economy and can be used to create a predictive I/O model for estimating economic impacts. IMPLAN uses both the industry x industry Input-Output table as well as the industry x commodity Input-Output table in the process of calculating the multipliers and impacts.

In this case, the direct investment from the NIH to the FCHES is plugged into the model as the input and the model specifications and assumptions are used to estimate how that money is spent in Genesee county. This spending on goods and services has a ripple effect in the economy (i.e. multipliers) and the model quantifies the degree to which other sectors and persons in Genesee county benefit from the initial investment. Using data on the spending patterns of 536 individual industry sectors (e.g. different types of farming, construction, manufacturing, and services), we were able to model the cross-sector economic activity that occurred throughout Genesee County as a result of the FCHES investment.

### I/O model specification

Several considerations went into the IMPLAN model specification. First, due to the nature of the NIMHD investment in research, we selected a single industry sector - Scientific Research and Development Services (IMPLAN sector number 456) – to estimate these economic impacts. FCHES’ mission is to conduct and facilitate scientific research. FCHES is not a provider of health services (e.g. clinical, inpatient, services). As a result, the Scientific Research and Development Services is the most relevant sector for our analyses, as opposed to health service sectors such as Hospitals or Physician’s Offices (IMPLAN sector numbers 490 and 483, respectively). Second, our model uses the 2016 year of economic data for the NIMHD investment in 2016, and the 2017 data year for each subsequent annual investment (2017–2020). This was a limitation of the model because the economic data for years 2018 and beyond were not available at the time of analysis. Third, because of the nature of scientific research centers and their difference from a typical company of which the owner receives profits, proprietor income is set to $0. Last, we used the actual employment, full time employee percentages (FTE%), and actual compensation of FCHES employees to increase the precision modeling the economic impact of FCHES.

### Data used

The funding amounts used as inputs in the I/O model reflect the actual funding amounts the center received, publicly available in the NIH Reporter system. The inputs included the initial investment as well as supplemental investments (NIH administrative supplements) by year. Compensation data was collected from the FCHES’ accounting department and includes all salaried, hourly, and consultant positions. In order to account for inflation and to represent all financial amounts in a common dollar year, all financial inputs were converted to the 2020 dollar year before being entered into the I/O model. This (vs not adjusting for inflation across time) allowed for the estimation of overall, multi-year, financial outputs of the IMPLAN model, and had no effect on the yearly impact multipliers due to the constant return to scale I/O assumption built into the IMPLAN model. Inflation and conversion to the 2020 dollar year were calculated using the U.S. Bureau of Labor Statistics’ yearly CPI data, which captures changes in the prices of all goods and services purchased for consumption, not specific to economic sectors. Data on the actual employment hours for hourly employees were converted to FTEs based on a 40 h work week. Salaried employees contributed one full time employment year per year of employment, and adjustments were made for hiring and employee turnover during the year.

### The IMPLAN I/O model

**Fig. 3:**
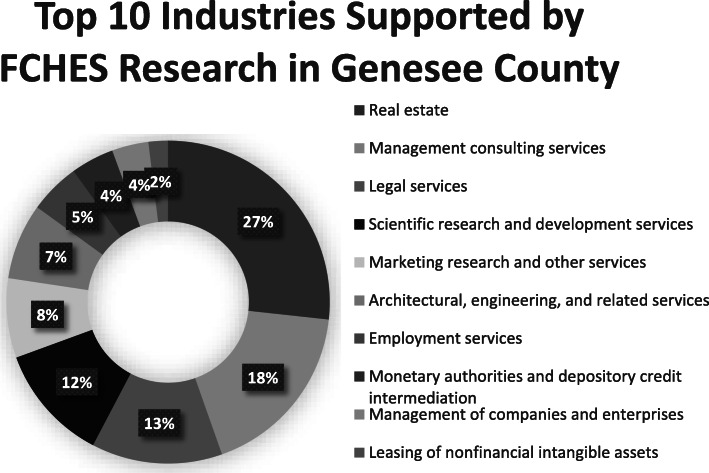
Cross-sectoral links between FCHES and other sectors of the local economy

What is commonly referred to as ‘economic impacts’ are calculated in IMPLAN as ‘Output’. The Output metric is composed of 5 categories that represent the ways in which value is added into the economy: 1. Intermediate Expenditures; 2. Employee Compensation; 3. Proprietor Income; 4. Taxes on Production and Imports; 5. Other Property Income. All of these are used to estimate the economic impact within the model. As the IMPLAN model incorporates 536 individual industry sectors, it allows for the modelling of cross-sector economic activity resulting from the FCHES work (Fig. 3). The overall economic impacts consist of three impact types: 1. Direct (the specific expenditures impact of FCHES and the sector in question); 2. Indirect (the impact on suppliers to FCHES and the Scientific Research and Development Services sector); and 3. Induced (the additional economic impact of the spending of these suppliers and employees in the economy). Built into the model, IMPLAN uses both the industry x industry I/O table as well as the industry x commodity I/O table in the process of calculating the multipliers and impacts.

**Fig. 4:**
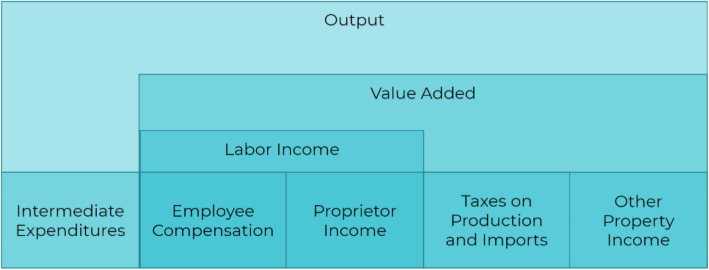
The structure of the IMPLAN economic model (graphic provided by [10])

The following results are provided for each impact estimation (see Fig. 4): employment, measured in full time job-years; personal income, including both wages and benefits; economic output; state and local tax revenue, including income and property taxes; and federal tax revenue, including contributions to Social Security. An impact multiplier is also provided for each type of data. Multipliers are calculated by dividing the total impact by the direct impact.

The FCHES research funding data includes the annual NIH federal investment in FCHES (Table 1). Table 2 reports the impact of 1 year (2019) of the FCHES funding to enable comparisons to other projects on a year-by-year basis. Table 3 (complete results available from the authors) reports the economic impact of the investment in the Flint Center for all individual years to assess stability of the FCHES economic impact over time. Table 4 reports the cumulative economic impact of the FCHES federal funding. All amounts are expressed in 2020 equivalent dollars.

**Table 1:** NIH annual investment in FCHES

	2016	2017	2018	2019	2020	Total
Initial Investment	$2,382,051	$2,195,822	$2,108,424	$2,124,467	$2,043,062	$10,853,827
Supplemental Investment	$0	$53,499	$110,049	$0	$0	$163,549
**Total**	$2,382,051	$2,249,322	$2,218,474	$2,124,467	$2,043,062	$11,017,376

**Table 2:** Annual Economic Impact of FCHES Federal Funding in 2019

Impact	Employment (Job-Years)	Personal Income	Output	State and Local Tax Revenue	Federal Tax Revenue
Direct Effect	21.17	$1,174,865	$2,124,467	$40,277	$246,234
Indirect Impacts	7.56	$330,930	$899,455	$37,926	$71,683
Induced Impacts	6.60	$270,289	$824,519	$52,928	$61,940
**Total Impact**	**35.33**	**$1,776,084**	**$3,848,441**	**$131,131**	**$379,857**
**Impact Multiplier**	**1.67**	**1.51**	**1.81**	**3.26**	**1.54**

**Table 3:** Summary of annual economic impacts, 2016–2020: Impact Multipliers

***Impact Multipliers***	***Employment (Job-Years)***	***Personal Income***	***Output***	***State and Local Tax Revenue***	***Federal Tax Revenue***
2016	2.45	2.12	1.61	3.49	2.09
2017	1.66	1.65	1.72	3.32	1.68
2018	1.65	1.56	1.78	3.28	1.59
2019	1.67	1.51	1.81	3.26	1.54
2020	1.63	1.49	1.83	3.24	1.52

**Table 4:** Cumulative Economic Impact of FCHES Federal Funding, 2016–2020

Impact	Employment (Job-Years)	Personal Income	Output	State and Local Tax Revenue	Federal Tax Revenue
Direct Effect	93.43	$4,781,991	$11,017,376	$184,395	$1,019,039
Indirect Impacts	39.16	$1,700,495	$4,659,028	$194,354	$371,057
Induced Impacts	28.62	$1,161,465	$3,562,745	$231,201	$268,653
**Total Impact**	**161.21**	**$7,643,951**	**$19,239,150**	**$609,950**	**$1,658,749**
**Impact Multiplier**	**1.73**	**1.60**	**1.75**	**3.31**	**1.63**

## Results

The total NIH investment in FCHES exceeded $11 million (Table 1).

The $2.1 million investment in FCHES in 2019 was directly responsible for 21 jobs (expressed as years of full-time employment – job-years), over $1.1 million in personal income and over $286,000 in state/local and federal tax revenue (Table 2). Through the spending of the center, additional indirect impacts were generated in Genesee County, including more than 7 job-years, close to $331,000 personal income to employed persons, and almost $900,000 in additional economic output. In addition, the personal spending of the FCHES researchers and staff and of the employees in the indirectly created jobs generated induced impacts of close to 7 additional job-years, over $270,000 in personal income, and an economic output of over $800,000. Overall, combined direct, indirect, and induced impacts of one year of FCHES federal investment in Genesee County included over 35 job-years, close to $1.8 million in personal income, and more than $3.8 million in economic output. In addition, this combined economic activity generated over $500,000 in state/local and federal tax revenue.

Figure 3 illustrates the cross-sectoral links between FCHES and the top ten sectors of the economy supported by the FCHES research activities. For example, the IMPLAN data indicates that the largest cross-sectoral impacts of an additional investment in FCHES will be found in the real estate sector (27%), management consulting services (18%), and in the legal services sector (13%), among the top ten sectors impacted by FCHES.

The impact multipliers were remarkably stable across time (Table 3), with the exception of the first year, the startup year. For example, the employment impact multiplier was 2.45 in year 1, and ranged between 1.63–1.67 in years 2–5, while the output multiplier was 1.61 in year 1, and ranged between 1.72–1.83 in years 2–5. The reasons why, to some extent, year 1 was an outlier include a similar funding amount as in subsequent years and a reduced duration as funding was awarded late in the year, which resulted in substantially lower FTEs in the first year.

The over $11 million total federal investment in FCHES between 2016 and 2020 was directly responsible for 93 job-years, $4.8 million in personal income and over $1.2 million in state/local and federal tax revenue (Table 4). Additional indirect impacts were generated in Genesee County through the spending of the center, including 39 job-years, over $1.7 million personal income to employed persons, and close to $4.7 million in added economic output. In addition, the personal spending of the FCHES researchers and staff and of the employees in the indirectly created jobs generated induced impacts of close to 29 job-years, close to $1.2 million in personal income, and an economic output of over $3.5 million. Overall, combined direct, indirect, and induced impacts of the total FCHES federal investment in Genesee County included over 161 job-years, over $7.6 million in personal income, and more than $19.2 million in economic output. In addition, this combined economic activity generated close to $2.3 million in state/local and federal tax revenue. The impact multipliers show the ripple effect of the FCHES investment. For example, the overall output of over $19.2 million led to an impact multiplier of 1.75 – every $1 of federal FCHES investment led to an additional $.75 of economic output in Genesee County.

## Discussion

### Main study findings

Overall, combined direct, indirect, and induced impacts in Genesee County of the $11 million FCHES federal investment included over 161 job-years, over $7.6 million in personal income, and more than $19.2 million in economic output. In addition, this combined economic activity generated close to $2.3 million in state/local and federal tax revenue. The overall output of over $19.2 million led to an impact multiplier of 1.75 – every $1 of federal FCHES investment led to an additional $.75 of economic output in Genesee County. The linkages between FCHES and other sectors of the economy indicate a relatively broad range of cross-sectoral economic impacts of an additional investment in research.

### The findings in relation to other studies

This is the first study, to our knowledge, applying an I/O framework to estimate the economic impacts of a large multidisciplinary research center. The method may be relevant and generalizable to other TCCs or other large research centers such as FCHES. Our estimates of the FCHES economic impact are consistent with the few prior studies assessing the economic impact of investment in research. Tripp and Grueber [8] examined the economic impact of the Human Genome Project (HGP) using a similar impact estimation method as this study. They found that the cumulative economic impact of Human Genome Sequencing federal funding (1998–2003) led to an output impact multiplier of close to 3 at the national level. The study, which is the most similar to ours, used IMPLAN to estimate the impact of federal investment in the throughout the entire US economy, which explains the larger multiplier compared to our estimated impact in one county. Our estimated multipliers, including the 1.75 output impact multiplier, are smaller as the economic impacts in this study were estimated in only one Michigan county. The size of the region considered in an IMPLAN model affects the magnitude of the estimated impact multipliers. As the region considered expands, the amount of additional spending that stays within that region increases, resulting in larger economic multipliers. Chatterjee and DeVol [7], using a production function as the input-output framework, evaluated economic returns of NIH funding in the Biosciences industry. They estimated that the funding led to an estimated output impact multiplier of 1.70 in the bioscience industry, similar to the output multiplier estimated by this study. United for Medical Research [11] reported that in fiscal year 2018, total NIH research funds awarded in 50 states and D.C. was over $28 billion, with an estimated impact in total economic activity nationwide of close to $74 billion, which implies an impact multiplier of 2.63. Similar in size with the Tripp and Grueber [8] study, the estimated output multiplier was larger than the one estimated by our study largely due to the national scope of the estimated impact. Using different methodologies, and therefore not directly comparable to the findings of our study and the other US studies cited above, several studies estimated the economic returns of medical research in the UK [12–15] and Australia [16]. The UK studies estimated the rate of return in terms of broad economic benefits of 15%, implying a 1.15 impact multiplier, while the Australian report estimated the rate of return in terms of broad economic gains of 30%, implying an impact multiplier of 1.3.

To further put the findings in perspective, we re-estimated the impact and multipliers in a scenario where the same amount of funding would have been invested in the hospital sector in the same county as FCHES (results available from the authors). Compared to a hospital in the same county receiving identical funding, FCHES funding resulted in more people employed, more total economic output, with a higher impact multiplier (1.75 vs 1.63), and led to more state, local and federal taxes being paid. FCHES generates scientific research, not providing health services, so it may not be directly competing for funding with local hospitals and other health providers. However, this comparison may be useful for policy makers making decisions under budgetary constraints and having to decide whether to fund research vs funding health services.

### Functional impacts of FCHES

While the economic impact of FCHES was the focus of this study, the primary goal of FCHES is to address health disparities and chronic disease that cross boundaries and directly affect the Flint and Genesee County Community. These advancements in knowledge and practice are expected to benefit healthcare and health of the Flint and Genesee County population, and beyond. We refer to these benefits in the current study as *functional impacts*. The functional FCHES impacts include expanding the scientific knowledge by embracing broad and inclusive research themes, intervening to reduce physical and behavioral health disparities, improving methods in disparities research, improving practices through dissemination, developing networks of collaborators and stakeholders, increasing partner organization capacity to effectively participate in the research enterprise, and engaging and enhancing community residents awareness and participation in reducing health inequities.

As best examples of functional impacts, the FCHES research projects include the Church Challenge (CC) and Strengthening Flint Families (SFF). The Church Challenge objective is to support healthy living environments and improve the health of the Flint community, with a focus on effective blood pressure management. The CC was developed using community-based participatory approaches and is based on a church-based program developed by and for primarily African-American Flint church congregations. This multilevel intervention addresses health at the community (level 3), church (level 2), and individual (level 1) to reduce blood pressure, reduce chronic disease risk, and promote health equity and wellbeing in Flint. The CC RCT works within churches and with individuals and is designed to examine the effectiveness of a community-designed, community-based, multilevel physical activity and nutrition program relative to an enhanced treatment as usual - the Health and Wellness Program. By June 2020, the Church Challenge had increased food availability for 176 participants, connected 273 participants to local resources that support healthy living, and disseminated health improvement strategies through 21 churches.

Strengthening Flint Families is a community-based behavioral health intervention designed to reduce behavioral health disparities and improve family resilience in the Flint Community by providing multiple levels of peer support and family services. Strengthening Flint Families is comprised of three behavioral health interventions: at the Individual level: Peer Recovery Coaches (PRCs); at the Family level: Strengthening Families Program (SFP); and at the Community-level: a Multi-Media Campaign. Phase 1 of SFF consisted of conducting 87 organization assessments and 9 key information interviews to identify available resources, gaps in services, and barriers to accessing behavioral health services in Flint. Phase 2 of SFF is the implementation phase in which results from phase 1 are used to identify and implement referral sites, host sites, and adopter sites for the SFP and PRC services. SFF provided SFP referral information to 77 organizations and has established a dedicated phone line and email address for organizations and community members to make referrals to the SFP. At the individual level, 4 PRCs were certified and 2 focus groups were conducted with 10 local PRCs to better understand the community needs. At the family level, as of June 2020, 22 families were referred to the SFP and 4 cohorts of the SFP have been completed with 16 youth and 10 adults receiving the full SFP program. At the community level, SFF placed 3 bus bench ads in strategic locations in Flint in November 2019 to promote the SFP and general behavioral health and wellbeing. The Community Playlist radio show hosted by Kristen Senters Young (Community PI of SFF) has produced 98 episodes in the past year. The show promotes the SFP as well as provides additional positive messaging to the community on prevention, treatment, and recovery. Episodes average 22.8 plays on the SoundCloud platform with 193 plays as the highest number of plays for one episode. The SoundCloud platform has grown to 28 followers in the past few years. The SFF Facebook page has grown to 151 likes and 170 followers, the SFF Twitter page has grown to 61 followers, and the SFF Instagram page has grown to 91 followers in the past few years.

### Limitations and strengths

Limitations inherent to the use of IMPLAN and other I/O models pertaining to this analysis can be found primarily in the models assumptions. These include constant returns to scale [17], the assumption of no supply constraints [18], fixed input structure [17–19], and the static nature of the model [19]. Constant returns to scale implies that outputs and inputs are related in a strictly linear manner (I.e., if input increases by 10%, output increases by 10%). This assumption may not hold in the case when reaching a certain threshold of input may translate to a new paradigm in output, or if a significant change in technology occurs, although this is unlikely to be case for this study because of the relative short time span of our study. If this assumption was violated, it is impossible to assess whether the output and multipliers were under-estimated or over-estimated, which is a limitation of this study. The assumption of no supply constraints implies zero restrictions to raw materials and employment and is a limitation of most studies estimating the economic impact of research and development. There are no concerns about restrictions to raw materials in this type of public health research, however, given the degree requirements for scientific research and the potential scarcity of higher education degree attainment, employment supply is a realistic concern in county-specific analyses similar to this study. To overcome this, our study used data on actual employment hours and compensations, not estimates, therefore any supply constraints are already built into our estimates and are unlikely to be of concern in the present study. Fixed input structures, another assumption of the IMPLAN model, posits that changes in the economy will affect the industry’s output level but not the mix of commodities and services it requires to produce that output. The authors have no reason to believe this assumption may have been violated and, therefore, had limited to no impact on the results of the study. Another assumption is that the model is static, meaning that the changes occur immediately and changes over time are not incorporated into the model outputs. In reality, all changes are occurring over time, which is a limitation of this study. However, with additional economic impacts potentially occurring over time, this limitation likely resulted in a conservative estimate of the economic impact and the impact multipliers.

Additional limitations, which are beyond the scope of this study, include comparative analyses of how the economy would have been affected given alternative counterfactual scenarios (e.g., tax reductions), opportunity costs, and more basic arguments about what constitutes a benefit (e.g. salaries) vs. a cost [20]. Although we did complete one analysis of what the economic impact would have been if the funding went to the hospital sector, analyses which involve other areas of the country, the opportunity cost of the additional labor created, or focused on tax reductions are not possible with the data we had access to. Addressing what constitutes a cost vs. a benefit is an important conceptual issue in economics, however the I/O framework views the costs of running the center as an investment in the larger community which we distinguished as the direct effect, and the output effect is estimated and presented separately from salaries, jobs, and taxes paid. Ultimately, we are aware of no other method which would allow for the quantification of the macroeconomic effects of an investment in research that would be more appropriate for the data we used.

Strengths of our analyses include the fact that we used actual data on FCHES employment and compensation. As IMPLAN was designed to calculate the total I/O of an industry, it is unusual to have a complete set of employment and compensation data and therefore creates estimates of employment and compensation averages specific to the industry and the area under study. When we added actual FCHES employment and compensation to the model, we found that this not only significantly increased the precision of the model but increased the estimated overall impact of FCHES as well, mostly due to a higher number of job years created compared to the model’s estimated employment and compensation data. The larger impact in the specific model suggests that FCHES employs significantly more individuals per dollar of investment than the industry average in Genesee County.

## Conclusions

The FCHES research funding yields significant direct economic impacts above and beyond the direct NIH investment of $11 million. The total impact in Genesee County, MI, of the five-year NIH investment in FCHES was over $19 million, creating over 161 job-years, $7.6 million personal income, and close to $2.3 million in tax revenue. The impact was broad and included effects in several economic sectors. The estimated impact multipliers were stable across the years, which may be an argument of investment in FCHES and other similar large research centers since the effects are anticipated to be predictable. The study findings, including the positive return on investment resulting from the economic output, support the continued funding of medical research. The economic impact estimation method may be relevant and generalizable to other Transdisciplinary Collaborative Centers or other large research centers such as FCHES. In addition, this study may be useful to government officials seeking to understand the direct and contemporary benefits of government investment in health research. Looking into the future, upcoming studies will assess more broadly the effects of the investment in FCHES in the counties bordering Genesee, MI, other counties, and statewide in Michigan.

## Data Availability

The data that support the findings of this study are available from IMPLAN, LLC but restrictions apply to the availability of these data, which were used under license for the current study, and so are not publicly available. Output data from the IMPLAN model are however available from the authors upon reasonable request and with permission of IMPLAN, LLC.

## Abbreviations

NIMHD: National institute of minority health and health disparities
FCHES: Flint center for health equity solutions
I/O: Input/output
TCC: Transdisciplinary collaborative centers
NIH: National institutes for health
